# Prediction of surgical difficulty in minimally invasive surgery for rectal cancer by use of MRI pelvimetry

**DOI:** 10.1002/bjs5.50292

**Published:** 2020-04-28

**Authors:** T. Yamamoto, K. Kawada, Y. Kiyasu, Y. Itatani, R. Mizuno, K. Hida, Y. Sakai

**Affiliations:** ^1^ Department of Surgery, Graduate School of Medicine Kyoto University, 54 Shogoin‐Kawara‐cho, Sakyo‐ku Kyoto Japan 606‐8507

## Abstract

**Background:**

Technical difficulties in rectal surgery are often related to dissection in a limited surgical field. This study investigated the clinical value of MRI pelvimetry in the prediction of surgical difficulty associated with minimally invasive rectal surgery.

**Methods:**

Patients with rectal cancer who underwent laparoscopic or robotic total mesorectal excision between 2005 and 2017 were reviewed retrospectively and categorized according to surgical difficulty on the basis of duration of surgery, conversion to an open procedure, use of the transanal approach, postoperative hospital stay, blood loss and postoperative complications. Preoperative clinical and MRI‐related parameters were examined to develop a prediction model to estimate the extent of surgical difficulty, and to compare anastomotic leakage rates in the low‐ and high‐grade surgical difficulty groups. Prognosis was investigated by calculating overall and relapse‐free survival, and cumulative local and distant recurrence rates.

**Results:**

Of 121 patients analysed, 104 (86·0 per cent) were categorized into the low‐grade group and 17 (14·0 per cent) into the high‐grade group. Multivariable analysis indicated that high‐grade surgical difficulty was associated with a BMI above 25 kg/m^2^ (odds ratio (OR) 4·45, *P* = 0·033), tumour size 45 mm or more (OR 5·42, *P* = 0·042), anorectal angle 123° or more (OR 5·98, *P* = 0·028) and pelvic outlet less than 82·7 mm (OR 6·62, *P* = 0·048). All of these features were used to devise a four‐variable scoring model to predict surgical difficulty. In patients categorized as high grade for surgical difficulty, the anastomotic leakage rate was 53 per cent (9 of 17 patients), compared with 9·6 per cent (10 of 104) in the low‐grade group (*P* < 0·001). The high‐grade group had a significantly higher local recurrence rate than the low‐grade group (*P* = 0·002).

**Conclusion:**

This study highlights the impact of clinical variables and MRI pelvimetry in the prediction of surgical difficulty in minimally invasive rectal surgery.

## Graphical abstract

This study highlights the impact of MRI pelvimetry in predicting surgical difficulty in laparoscopic rectal surgery. Multivariable analysis identified that surgical difficulty was significantly associated with four variables: BMI, tumour size, anorectal angle and pelvic outlet. A novel scoring model using these four variables to predict surgical difficulty is proposed.

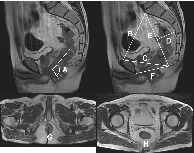

MRI pelvimetry and TME difficulty

## Abstract (es)

**Antecedentes:**

Las dificultades técnicas en la cirugía del recto a menudo están relacionadas con la disección en un campo quirúrgico limitado. Este estudio se propuso investigar la utilidad clínica de la pelvimetría con MRI para predecir la dificultad quirúrgica asociada con la cirugía mínimamente invasiva del recto.

**Métodos:**

Pacientes con cáncer de recto sometidos a resección total del mesorrecto laparoscópica o robótica entre 2005 y 2017 fueron revisados retrospectivamente y categorizados de acuerdo con la dificultad técnica en base al tiempo operatorio, conversión, uso de abordaje transanal, estancia hospitalaria postoperatoria, pérdida sanguínea y complicaciones postoperatorias. Se examinaron parámetros clínicos preoperatorios y relacionados con la MRI para desarrollar un modelo de predicción que estimara el grado de dificultad técnica y pudiera comparar la tasa de dehiscencias anastomóticas en los grupos de bajo y alto riesgo. También se evaluó el pronóstico con el cálculo de la supervivencia global, supervivencia libre de recidiva y tasas acumuladas de recidiva local y a distancia.

**Resultados:**

De un total de 121 pacientes analizados, 104 pacientes (85,9%) fueron categorizados en el grupo con bajo grado de dificultad técnica, mientras que 17 pacientes (14,1%) lo fueron en el grupo de alto grado. En el análisis multivariable, un alto grado de dificultad técnica se asociaba con un índice de masa corporal (*body mass index*, BMI) > 25 kg/m^2^ (razón de oportunidades, *odds ratio* (OR), 4,5; *P* = 0,033), tamaño tumoral ≥ 45 mm (OR, 5,4; *P* = 0,042), ángulo anorrectal ≥ 123 grados (OR, 6,0; *P* = 0,028), estrecho pélvico < 82,7 mm (OR, 6,6; *P* = 0,048); en consecuencia, todas estas características fueron utilizadas para establecer un modelo de puntuación con 4 variables que pudiera predecir la dificultad técnica. En los pacientes categorizados como de alto grado de dificultad técnica, la tasa de dehiscencia anastomótica fue del 52,9%, mientras que en el grupo de bajo grado fue del 9,6% (*P* < 0,001). Finalmente, el grupo con alto grado mostró una tasa significativamente más elevada de recidiva local en comparación con el grupo de bajo grado (*P* = 0,002).

**Conclusión:**

Este estudio pone de relieve el impacto de variables clínicas y de la pelvimetría con MRI para predecir la dificultad técnica en la cirugía mínimamente invasiva del recto.

## Introduction

Total mesorectal excision (TME) is the standard technique for rectal surgery^1^ and the quality of the surgical technique directly affects postoperative local recurrence. In addition to the surgeon's skill, surgical safety and quality depend on patient‐related clinical and anatomical factors. BMI, sex, tumour height, tumour size, and dimensions of the pelvic cavity are well known predictive factors of surgical difficulty in patients undergoing rectal surgery. TME is challenging in patients with a narrow and deep pelvis because the bony structure surrounding the rectum disturbs the surgical manoeuvre. Limited pelvic space can cause insufficient countertraction, leading to incomplete TME.

Compared with open surgery, some authors^2^, ^3^ have proposed that laparoscopic rectal surgery can improve the quality of TME as a result of better visualization of the surgical field. However, retraction of pelvic structures and rectal transection may be more difficult owing to the narrow pelvic space. Although widely accepted, the definitive role of laparoscopic surgery in treatment of rectal cancer remains controversial. Two recent RCTs, the COLOR II^4^ and COREAN^5^ trials, reported an advantage for laparoscopic rectal resection compared with the open approach, whereas the ALaCaRT^6^ and ACOSOG Z6051^7^ trials failed to prove the non‐inferiority of minimally invasive surgery. This controversy can be attributed to the technical difficulty associated with laparoscopic rectal surgery.

MRI is a necessary tool in devising the optimal treatment strategy for rectal cancer by defining the tumour depth, circumferential resection margin (CRM) involvement, extramural vascular invasion and lateral pelvic lymph node enlargement^8^, ^9^. In addition, MRI is useful in surgical planning by assessing the pelvic shape and positional relationship of anatomical landmarks. Several anatomical parameters, such as size of the pelvis, prominence of the sacral promontory and degree of sacral curvature, are associated with technical difficulties in performing rectal surgery; however, it remains debatable whether MRI pelvimetry can predict the extent of surgical difficulty.

The present study aimed to measure the anatomical variables on MRI, and to investigate their predictive value in estimating the surgical difficulty associated with minimally invasive rectal surgery.

## Methods

Patients with locally advanced rectal cancer (within 10 cm of the anal verge) who had preoperative MRI and laparoscopic or robotic low anterior resection (LAR) or intersphincteric resection (ISR) at Kyoto University Hospital (volume of rectal cancer procedures 60 per year) between July 2005 and June 2017 were enrolled. Patients who underwent abdominoperineal resection (APR) or extended surgery (such as combined resection of prostate or liver) were excluded. Patients undergoing transanal TME surgery were also excluded to address the surgical difficulty of TME from the transabdominal approach.

Based on the English National Low Rectal Cancer Development Programme definition on T2‐weighted MRI scans^10^, tumours located below the line between the pubic bone and the origin of the levator ani muscles were categorized as low, whereas those located above this line were categorized as high. All patients were categorized as having UICC TNM stage II–III disease, and those with stage IV disease were excluded. Tumour size and distance from the anal verge were measured using T2‐weighted MRI scans in the sagittal plane. Preoperative treatment, such as neoadjuvant chemoradiotherapy (nCRT: 45 Gy in 25 fractions with concomitant S‐1 and irinotecan) or neoadjuvant chemotherapy (NAC: modified FOLFOX6, FOLFIRI, or S‐1 plus irinotecan), was administered to the patients with a high risk of recurrence (such as those with bulky tumours or marked lymph node enlargement)^11^. Clinical variables collected included age, sex, BMI, clinical stage, carcinoembryonic antigen (CEA) level, tumour location, surgical procedure, blood loss, duration of surgery, postoperative morbidity and postoperative hospital stay.

The study protocol was approved by the Institutional Review Board of Kyoto University (reference number R1957).

### MRI pelvimetry

**Figure 1:**
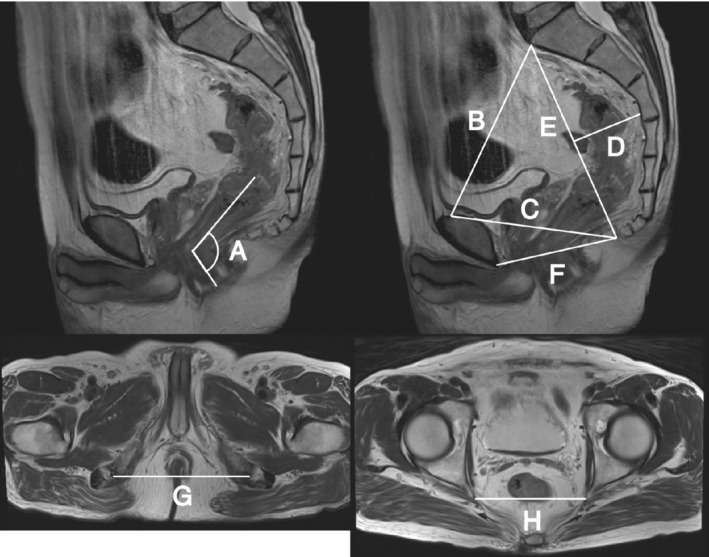
MRI pelvimetry A, anorectal angle (angle between anal canal and rectum). B, pelvic inlet (distance between sacral promontory and superior aspect of pubic symphysis). C, pubococcygeal distance (distance between tip of the coccyx and superior aspect of pubic symphysis). D, sacral depth (perpendicular distance from deepest portion of the sacrococcygeal hollow to the sacrococcygeal line). E, pelvic length (distance between sacral promontory and tip of the coccyx). F, pelvic outlet (distance between tip of the coccyx and inferior aspect of pubic symphysis). G, intertuberous distance (distance between the lowest points of the ischial tuberosities). H, interspinous distance (distance between tips of the ischial spines).

All patients enrolled in the present study had abdominal MRI within 3 weeks before surgery. The following eight MRI pelvimetric parameters were measured in each patient using T2‐weighted MRI scans (*Fig*. 1): anorectal angle (angle between anal canal and rectum); pelvic inlet (distance between sacral promontory and superior aspect of pubic symphysis); pubococcygeal distance (distance from the tip of the coccyx to the superior aspect of the pubic symphysis); sacral depth (perpendicular distance from the deepest portion of the sacrococcygeal hollow to the sacrococcygeal line); pelvic length (distance between sacral promontory and the tip of the coccyx); pelvic outlet (distance between the tip of the coccyx and the inferior aspect of the pubic symphysis); intertuberous distance (distance between the lowest points of the ischial tuberosities); and interspinous distance (distance between tips of the ischial spines). The first six parameters were measured in the sagittal plane, and the last two in the axial plane. All measurements were recorded by two observers who were blinded to the clinical data. Reanalysis was performed to draw a definitive conclusion where there was more than 5 per cent interobserver difference in the results.

### Surgical procedure

All surgical procedures were performed by board‐certified laparoscopic colorectal surgeons^12^ using a standard TME technique. In patients who had LAR, an end‐to‐end double‐stapling technique anastomosis was completed intracorporeally after rectal transection using a linear stapler^13^. In patients who underwent ISR, an initial abdominal approach was used, as low as possible to the pelvic floor, followed by intersphincteric dissection under direct vision with creation of a handsewn end‐to‐end coloanal anastomosis^14^. Lateral pelvic lymph node dissection was done selectively in patients with suspected metastatic lateral pelvic lymph nodes (lymph nodes measuring 5·0 mm or more in short‐axis diameter)^9^.

### Outcome measures

The criteria used to assess surgical difficulty were based on the previously published data^15^, ^16^, ^17^, ^18^. Briefly, the following six criteria were used to measure surgical difficulty associated with rectal surgery: duration of surgery longer than 350 min; conversion to open surgery; additional use of the transanal approach (difficult cases in which the transanal approach was required in addition to a transabdominal approach to complete TME); duration of postoperative hospital stay greater than 18 days; intraoperative blood loss above 100 ml and postoperative complications with Clavien–Dindo^19^ grade II or above. The surgical difficulty score was estimated using these six criteria, with scores ranging between 0 and 12 points (*Table* *S1*, supporting information)^15^.

Based on the surgical difficulty score, patients were classified into two groups; patients with fewer than 6 points (low‐grade group) and those with a score of 6 points or more (high‐grade group). Clinical variables correlated to the surgical difficulty were analysed, as well as the effect of the surgical difficulty score on prognostic outcome.

Clinical and MRI variables were analysed to calculate a prediction model and to compare the rate of anastomotic leakage (defined as extravasation of endoluminally administered contrast enema and fluid/air bubbles surrounding the anastomosis on radiographic examination) in low‐ and high‐grade groups.

Prognostic outcomes were evaluated by calculating the overall survival (OS), defined as the time from the date of the initial surgery to the date of death from any cause, relapse‐free survival (RFS), defined as the time from the date of the initial surgery to the date of cancer recurrence or the date of death from any cause, the cumulative local recurrence rate (the rate of pelvic cavity recurrence estimated by the Kaplan–Meier method), and the cumulative distant recurrence rate (the rate of distant metastasis estimated by the Kaplan–Meier method).

Follow‐up was conducted as follows: blood tests and physical examination were performed every 3 months for the first 3 years and every 6 months thereafter; CT was performed every 3 months (for stage III disease) or every 6 months (for stage II disease) in the first 3 years, then every 6 months for at least 2 years; and colonoscopy was performed at 1, 3 and 5 years after surgery.

The impact of preoperative treatment on MRI‐related parameters was also evaluated, in relation to tumour size and MRI pelvimetric parameters.

### Statistical analysis

Continuous variables are presented as median (i.q.r.) values. The χ^2^ or Fisher's exact test was used to determine the association between preoperative data and surgical difficulty score for categorical variables, and the Mann–Whitney *U* test for continuous variables. All criteria were used to create an in‐house surgical difficulty score, referring to the respective median values or clinical implications.

Multivariable logistic regression analysis was performed to determine the predictors of high‐grade surgical difficulty, including clinical factors with *P* ≤ 0·100 and MRI pelvimetric parameters. The cut‐off values for MRI pelvimetric parameters were determined as the respective median values. A prediction model using a logistic regression coefficient was employed to calculate a prediction score based on this formula, assigning 1 point to each variable; the scoring model was proposed with the use of sum total in points.

OS, RFS and cumulative recurrence rates were evaluated using the Kaplan–Meier method and log rank test. All tests were two‐sided, conducted with JMP® Pro version 14 (SAS Institute, Cary, North Carolina, USA), and a *P* < 0·050 was considered statistically significant.

## Results

Of 240 patients who had curative resection for stage II–III rectal cancer during the study period^11^, 121 patients who had preoperative MRI were analysed. *Table* 1 shows the clinicopathological characteristics of these patients, including 82 men and 39 women with a median age of 65 years. LAR was performed in 107 patients, and ISR was done in 14. The conversion rate to open surgery was 2·5 per cent (3 of 121). Lateral pelvic node dissection was performed in 16 patients with clinical suspicion of metastatic lateral pelvic nodes. nCRT was employed in eight patients, and NAC in 32. Thirty‐seven patients who received preoperative treatment (nCRT or NAC) had abdominal MRI before and after this treatment. Postoperative complications categorized as Clavien–Dindo grade II or above occurred in 26 patients, but no mortality was observed.

**Table 1:** Clinicopathological characteristics

	No. of patients* (*n* = 121)
**Age (years)** †	65 (56–71)
**Sex ratio (M** : **F)**	82 : 39
**BMI (kg/m** ^**2**^ **)** †	22 (19·3–24·0)
**Preoperative treatment**	
None	81 (66·9)
Chemotherapy	32 (26·4)
Chemoradiotherapy	8 (6·6)
**cT category**	
cT1	2 (1·7)
cT2	11 (9·1)
cT3	89 (73·6)
cT4	19 (15·7)
**CEA (ng/ml)** †	4·0 (2·2–10·6)
**Tumour location**	
High	71 (58·7)
Low	50 (41·3)
**Surgical procedure**	
LAR	107 (88·4)
ISR	14 (11·6)
**LPLND**	16 (13·2)
**Surgical technique**	
Laparoscopic	101 (83·5)
Robotic	17 (14·0)
Conversion to open operation	3 (2·5)
**Additional use of transanal approach**	1 (0·8)
**Resection margin**	
R0	120 (99·2)
R1	1 (0·8)
**Blood loss** †	30 (10–85)
**Duration of surgery (min)** †	310 (268–388)
**Postoperative hospital stay (days)** †	18 (14–26)
**Morbidity ≥ grade II**	26 (21·5)
**Tumour size on MRI (mm)** †	45·1 (36·9–54·2)
**Tumour height from anal verge on MRI (mm)** †	67·4 (52·8–80·9)
**MRI pelvimetry data** †	
Anorectal angle (°)	123·1 (115·6–135·6)
Pelvic inlet (mm)	117·1 (108·8–123·9)
Pubococcygeal distance (mm)	107·9 (102·4–114·0)
Sacral depth (mm)	37·8 (33·3–42·7)
Pelvic length (mm)	125·6 (114·9–133·6)
Pelvic outlet (mm)	82·7 (78·1–87·7)
Intertuberous distance (mm)	103·6 (93·6–112·6)
Interspinous distance (mm)	94·2 (87·5–104·0)

* With percentages in parentheses unless indicated otherwise;

† values are median (i.q.r.). CEA, carcinoembryonic antigen; LAR, low anterior resection; ISR, intersphincteric resection; LPLND, lateral pelvic lymph node dissection.

Reanalysis of the eight pelvic parameters measured using MRI pelvimetry (*Fig*. 1) was required in ten patients (8·3 per cent) owing to interobserver difference greater than 5 per cent.

### Surgical difficulty score

The parameters used to define the surgical difficulty score were associated closely with patient characteristics (*Table S2*, supporting information). Male sex was significantly associated with duration of surgery, length of hospital stay, intraoperative blood loss and postoperative morbidity. High BMI (above 25 kg/m^2^) was significantly associated with duration of surgery, hospital stay and blood loss during resection. Low tumour height was significantly associated with duration of surgery. An additional use of the transanal approach was required in only one patient, so this variable was excluded from analysis.

The associations between the MRI pelvimetry data and surgical difficulty criteria are shown in *Tables* 2, 3, 4, 5, 6. The anorectal angle was significantly associated with duration of surgery, blood loss and hospital stay. Pelvic length was significantly associated with duration of surgery and length of hospital stay. Intertuberous and interspinous distances were significantly associated with duration of surgery and intraoperative blood loss. The pubococcygeal distance was significantly associated with postoperative morbidity.

**Table 2:** Association between duration of surgery and MRI pelvimetry criteria

	Duration of surgery (min)		
	≤ 350	> 350	P*
**Tumour size (mm)**	43·8 (37·5–53·0)	48·1 (36·3–60·1)	0·363
**Tumour height from anal verge (mm)**	68·7 (56·0–82·3)	64·4 (52·6–71·9)	0·070
**MRI pelvimetry data**			
Anorectal angle (°)	119·0 (112·3–131·0)	132·1 (120·4–139·6)	0·002
Pelvic inlet (mm)	117·7 (108·7–124·0)	116·3 (109·1–123·6)	0·716
Pubococcygeal distance (mm)	106·5 (101·6–113·4)	109·8 (103·9–115·3)	0·192
Sacral depth (mm)	37·8 (33·6–43·5)	37·7 (33·0–42·5)	0·828
Pelvic length (mm)	124·5 (113·1–130·5)	127·5 (120·2–137·7)	0·040
Pelvic outlet (mm)	83·0 (76·5–87·5)	82·6 (79·4–89·2)	0·477
Intertuberous distance (mm)	107·2 (97·9–114·9)	99·9 (90·9–106·7)	0·005
Interspinous distance (mm)	95·4 (88·9–106·1)	92·4 (84·9–99·6)	0·043

Values are median (i.q.r.).

* Mann–Whitney *U* test.

**Table 3:** Association between blood loss and MRI pelvimetry criteria

	Blood loss (ml)		
	≤ 100	> 100	*P* *
**Tumour size (mm)**	43·3 (36·4–52·1)	54·1 (40·6–65·5)	0·023
**Tumour height from anal verge (mm)**	67·8 (54·0–80·9)	58·1 (51·5–84·7)	0·409
**MRI pelvimetry data**			
Anorectal angle (°)	120·4 (114·9–132·3)	135·3 (122·4–142·7)	0·006
Pelvic inlet (mm)	117·4 (109·0–123·7)	116·0 (107·2–124·5)	0·810
Pubococcygeal distance (mm)	107·7 (102·3–113·8)	111·3 (104·0–114·6)	0·445
Sacral depth (mm)	38·1 (33·5–43·0)	36·7 (33·1–41·6)	0·581
Pelvic length (mm)	125·6 (114·3–133·9)	125·8 (118·0–133·4)	0·507
Pelvic outlet (mm)	83·0 (77·8–87·5)	82·5 (78·2–89·2)	0·929
Intertuberous distance (mm)	106·1 (98·3–113·2)	94·8 (88·5–105·6)	0·001
Interspinous distance (mm)	95·4 (89·0–105·4)	89·2 (83·9–95·3)	0·021

Values are median (i.q.r.).

* Mann–Whitney *U* test.

**Table 4:** Association between conversion and MRI pelvimetry criteria

	Conversion		
	No	Yes	*P* *
**Tumour size (mm)**	45·0 (37·1–54·1)	58·5 (35·4–69·7)	0·360
**Tumour height from anal verge (mm)**	67·6 (52·9–81·2)	59·4 (50·3–67·2)	0·338
**MRI pelvimetry data**			
Anorectal angle (°)	123·3 (115·6–135·6)	122·4 (117·9–136·0)	0·861
Pelvic inlet (mm)	117·0 (108·7–123·7)	126·4 (116·9–136·2)	0·106
Pubococcygeal distance (mm)	107·9 (102·3–113·4)	118·5 (104·1–126·1)	0·164
Sacral depth (mm)	37·8 (33·3–42·6)	37·8 (25·6–46·5)	0·914
Pelvic length (mm)	125·6 (114·7–132·8)	142·0 (124·3–142·9)	0·138
Pelvic outlet (mm)	82·9 (78·1–87·7)	82·6 (76·8–102·3)	0·726
Intertuberous distance (mm)	103·6 (93·1–112·6)	106·6 (98·5–125·8)	0·505
Interspinous distance (mm)	94·1 (87·3–103·3)	105·2 (92·4–121·4)	0·191

Values are median (i.q.r.).

* Mann–Whitney *U* test.

**Table 5:** Association between length of hospital stay and MRI pelvimetry criteria

	Hospital stay (days)		
	≤ 18	> 18	*P* *
**Tumour size (mm)**	45·2 (32·1–53·1)	44·6 (39·1–58·9)	0·408
**Tumour height from anal verge (mm)**	68·7 (61·7–81·8)	61·1 (49·7–77·1)	0·050
**MRI pelvimetry data**			
Anorectal angle (°)	119·3 (112·1–132·3)	128·9 (117·9–136·5)	0·013
Pelvic inlet (mm)	118·8 (108·7–124·8)	117·0 (109·0–122·7)	0·719
Pubococcygeal distance (mm)	107·2 (102·0–112·5)	109·0 (103·9–115·5)	0·240
Sacral depth (mm)	38·0 (34·0–42·6)	37·8 (32·5–43·0)	0·485
Pelvic length (mm)	123·1 (112·3–130·4)	128·1 (119·3–137·2)	0·019
Pelvic outlet (mm)	83·0 (77·0–88·1)	82·6 (78·1–87·0)	0·849
Intertuberous distance (mm)	103·3 (94·1–115·5)	104·2 (93·0–110·3)	0·731
Interspinous distance (mm)	93·8 (88·6–106·4)	94·9 (86·3–100·5)	0·531

Values are median (i.q.r.).

* Mann–Whitney *U* test.

**Table 6:** Association between morbidity and MRI pelvimetry criteria

	Morbidity		
	No	Yes	*P* *
**Tumour size (mm)**	43·4 (33·4–53·1)	49·1 (40·8–62·4)	0·032
**Tumour height from anal verge (mm)**	66·5 (52·9–81·0)	69·6 (52·7–81·6)	0·575
**MRI pelvimetry data**			
Anorectal angle (°)	122·9 (115·0–135·1)	127·3 (117·9–136·1)	0·206
Pelvic inlet (mm)	117·8 (109·1–123·9)	115·4 (107·6–124·2)	0·466
Pubococcygeal distance (mm)	106·5 (102·2–112·6)	111·7 (105·6–116·6)	0·034
Sacral depth (mm)	38·3 (33·8–42·9)	34·8 (30·8–42·3)	0·204
Pelvic length (mm)	124·1 (113·0–133·4)	128·2 (121·4–134·8)	0·132
Pelvic outlet (mm)	82·3 (76·0–87·6)	84·0 (80·2–89·3)	0·098
Intertuberous distance (mm)	104·2 (94·8–113·5)	101·3 (92·1–108·2)	0·128
Interspinous distance (mm)	94·3 (88·7–105·9)	91·8 (84·5–101·1)	0·131

Values are median (i.q.r.).

* Mann–Whitney *U* test.

Based on the surgical difficulty score, 104 patients (86·0 per cent) had a score of less than 6 points (low‐grade group) and 17 (14·0 per cent) had 6 points or more (high‐grade group).

### Risk factors related to surgical difficulty

In univariable analysis, high grade of surgical difficulty was significantly associated with high BMI (above 25 kg/m^2^), serum CEA level and anorectal angle (*Table* 7).

**Table 7:** Univariable analysis for comparison of clinical characteristics and MRI pelvimetry parameters in low‐ and high‐grade groups

	Low‐grade group (*n* = 104)	High‐grade group (*n* = 17)	*P* †
**Age (years)**			
≤ 65	56 (53·8)	10 (59)	
> 65	48 (46·2)	7 (41)	0·702
**Sex**			
M	67 (64·4)	15 (88)	
F	37 (35·6)	2 (12)	0·056
**BMI (kg/m** ^**2**^ **)**			
≤ 25	89 (85·6)	9 (53)	
> 25	15 (14·4)	8 (47)	0·002
**CEA (ng/ml)** *	3·5 (2·0–8·3)	12·1 (4·5–14·9)	0·002‡
**Tumour location**			
High	63 (60·6)	8 (47)	
Low	41 (39·4)	9 (53)	0·294
**Anastomosis**			
LAR	93 (89·4)	14 (82)	
ISR	11 (10·6)	3 (18)	0·415
**cT category**			
cT1–2	12 (11·5)	1 (6)	
cT3–4	92 (88·5)	16 (94)	0·691
**Tumour size on MRI (mm)** *	43·5 (36·4–53·1)	50·9 (43·1–62·8)	0·057‡
**Tumour height from anal verge on MRI (mm)** *	67·7 (55·9–81·6)	58·1 (50·9–73·2)	0·141‡
**MRI pelvimetry data** *			
Anorectal angle (°)	120·5 (114·3–131·5)	136·0 (128·6–143·1)	0·001‡
Pelvic inlet (mm)	117·7 (108·8–123·7)	111·3 (108·3–128·6)	0·899‡
Pubococcygeal distance (mm)	107·8 (102·2–114·2)	109·4 (104·0–115·0)	0·514‡
Sacral depth (mm)	38·1 (33·7–42·8)	35·1 (31·6–43·1)	0·536‡
Pelvic length (mm)	125·4 (113·1–132·4)	127·2 (123·6–139·2)	0·093‡
Pelvic outlet (mm)	83·0 (76·9–87·6)	82·5 (78·8–88·9)	0·682‡
Intertuberous distance (mm)	104·6 (96·8–113·0)	98·5 (88·8–106·5)	0·057‡
Interspinous distance (mm)	94·3 (88·6–104·9)	92·4 (82·6–103·7)	0·451‡

Values in parentheses are percentages unless indicated otherwise;

* values are median (i.q.r.). CEA, carcinoembryonic antigen; LAR, low anterior resection; ISR, intersphincteric resection.

† χ^2^ or Fisher's exact test, except

‡ Mann–Whitney *U* test.

Multivariable analysis revealed that high grade of surgical difficulty was significantly associated with BMI above 25 kg/m^2^ (odds ratio (OR) 4·45, 95 per cent c.i. 1·13 to 17·54; *P* = 0·033), tumour size 45 mm or greater (OR 5·42, 1·06 to 27·64; *P* = 0·042), anorectal angle of 123° or more (OR 5·98, 1·22 to 29·34; *P* = 0·028) and pelvic outlet less than 82·7 mm (OR 6·62, 1·02 to 43·02, *P* = 0·048) (*Table* 8).

**Table 8:** Multivariable logistic regression analysis of predictors of surgical difficulty

	Odds ratio	*P*
Male sex	1·37 (0·14, 13·08)	0·782
BMI > 25 kg/m^2^	4·45 (1·13, 17·54)	0·033
CEA > 5·0 ng/ml	1·06 (0·26, 4·32)	0·934
Tumour size ≥ 45 mm	5·42 (1·06, 27·64)	0·042
Anorectal angle ≥ 123°	5·98 (1·22, 29·34)	0·028
Pelvic inlet < 117·1 mm	1·69 (0·39, 7·26)	0·482
Pubococcygeal distance < 108·0 mm	1·28 (0·29, 5·70)	0·750
Sacral depth < 37·8 mm	3·38 (0·60, 19·16)	0·169
Pelvic length ≥ 125·6 mm	1·25 (0·29, 5·42)	0·764
Pelvic outlet < 82·7 mm	6·62 (1·02, 43·02)	0·048
Intertuberous distance < 103·6 mm	1·07 (0·21, 5·32)	0·936
Interspinous distance < 94·2 mm	1·72 (0·39, 7·68)	0·476

Values in parentheses are 95 per cent confidence intervals. CEA, carcinoembryonic antigen.

### Prediction model for surgical difficulty and anastomotic leakage

By using the four variables associated with high grade of surgical difficulty in multivariable analysis, a logistic regression coefficient was employed to create the estimated formula: high risk = 0·93 × BMI + 1·05 × tumour size +1·11 × anorectal angle +1·17 × pelvic outlet.

**Figure 2:**
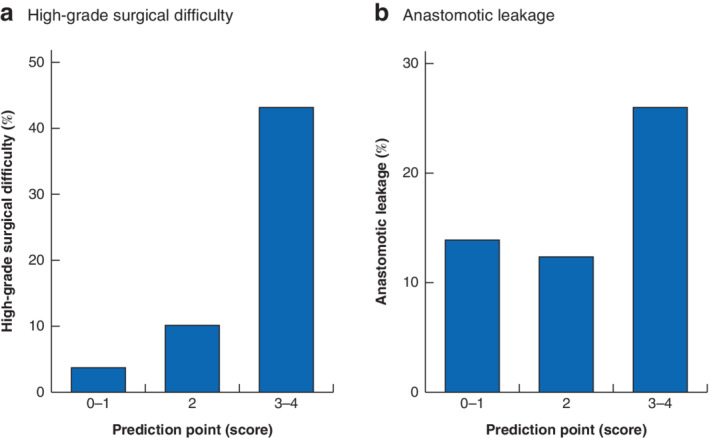
High‐grade surgical difficulty and anastomotic leakage according to the four‐variable scoring model **a** Proportion of patients assigned to the high‐grade surgical difficulty category, and **b** proportion of patients with anastomotic leakage in three groups created from the scoring model: patients with total points of 0–1 (*n* = 50); patients with 2 points (*n* = 48); and patients with 3–4 points (*n* = 23).

A simple prediction score for surgical difficulty was created from this formula. One point was assigned to each variable; a four‐variable scoring model was proposed with the use of the sum total in points (ranging from 0 to 4 points). Based on this scoring model, patients were classified into three groups: patients with total points of 0–1 (50 patients); patients with 2 points (48); and patients with 3–4 points (23). The proportions of patients assigned to the high‐grade category (with a score of 6 or more points) in the three groups were 4 per cent (2 of 50), 10 per cent (5 of 48) and 43 per cent (10 of 23) respectively (*Fig*. 2 *a*; *Table S3*, supporting information). In the whole cohort of 121 patients, anastomotic leakage occurred in 19 patients (15·7 per cent). The rates of anastomotic leakage in the three groups were 14 per cent (7 of 50), 13 per cent (6 of 48) and 26 per cent (6 of 23) respectively (*Fig*. 2 *b*).

**Figure 3:**
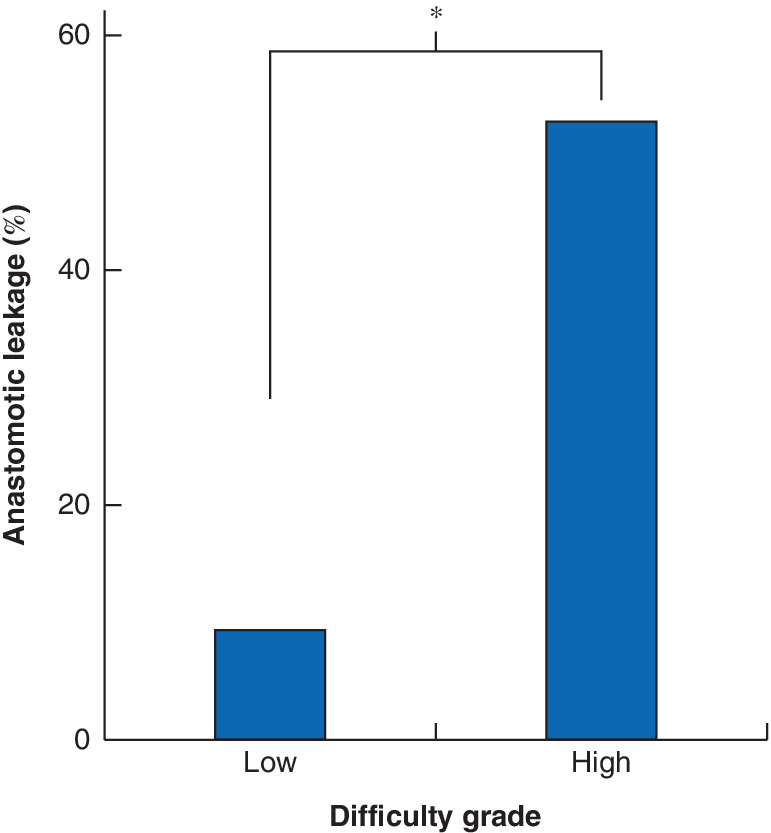
Rate of anastomotic leakage in high‐ and low‐grade surgical difficulty groups There were 104 patients in the group with low‐grade surgical difficulty and 17 in the group with high‐grade difficulty. *P < 0·001 (Fisher's exact test).

The relationship between anastomotic leakage and surgical difficulty was analysed. The leakage rate was 53 per cent (9 of 17) in patients categorized as high grade for surgical difficulty, whereas that in the low‐grade group was 9·6 per cent (10 of 104) (*P* < 0·001) (*Fig*. 3).

### Effects of surgical difficulty score on prognostic outcome

**Figure 4:**
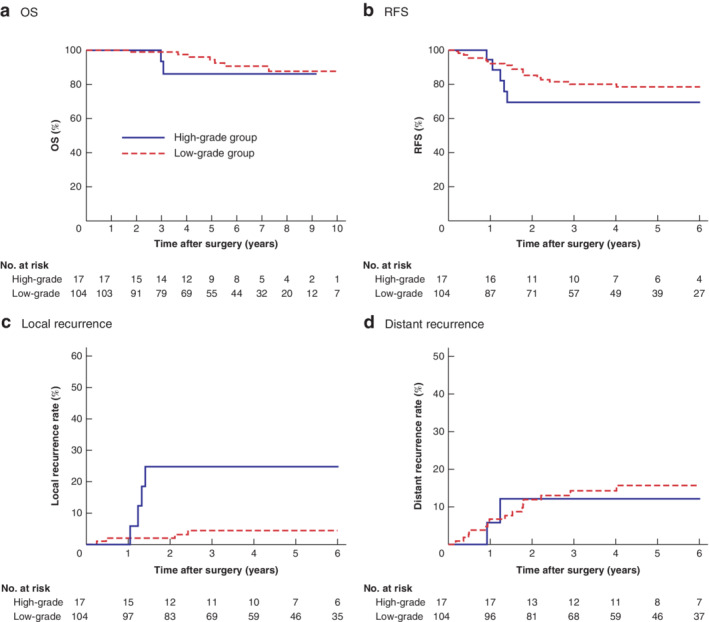
Kaplan–Meier analysis of overall survival, relapse‐free survival, and local and distant recurrence rates in high‐ and low‐grade surgical difficulty groups **a** Overall survival (OS); **b** relapse‐free survival (RFS); **c** cumulative rate of local recurrence; **d** cumulative rate of distant recurrence. **a** *P* = 0·526, **b** *P* = 0·320, **c** *P* = 0·002, **d** *P* = 0·769 (log rank test).

The median (range) duration of follow‐up was 62 (8–152) months. The association between surgical difficulty grade and patients' long‐term prognosis was also analysed (*Fig*. 4). Kaplan–Meier curves indicated that patients in the high‐grade group had a significantly higher local recurrence rate than those in the low‐grade group (*P* = 0·002) (*Fig*. 4 *c*). The 5‐year cumulative local recurrence rate was 25 per cent in the high‐grade group and 4·4 per cent in the low‐grade group. There was no significant difference between the two groups in OS, RFS or cumulative distant recurrence rates.

### Impact of preoperative treatment on MRI‐related parameters

The changes in MRI pelvimetric parameters in 37 patients who had abdominal MRI before and after preoperative treatment (NAC, 30; nCRT, 7) were determined. Tumour size decreased significantly after preoperative treatment (median 42·0 mm *versus* 60·4 mm before treatment; *P* < 0·001). Among the MRI pelvimetric parameters, only anorectal angle was significantly reduced after preoperative treatment (median 123·0° *versus* 134·7° before treatment; *P* = 0.008) (*Fig*. *S1*, supporting information).

## Discussion

Previous studies identified several variables to predict the difficulty of rectal resections. Sex, BMI, tumour size and location, nCRT and co‐morbidity, anastomotic height, histopathological features, surgeon experience and anatomical dimensions of the pelvis were reported to be risk factors for conversion to open surgery, duration of surgery, CRM positivity and postoperative morbidity^15^, ^16^, ^17^, ^18^, ^20^, ^21^, ^22^, ^23^, ^24^. Several MRI pelvimetric parameters have been investigated previously; however, the results were inconsistent owing to differences in sample size, the racial composition of patient populations, variables measured, and definitions of surgical difficulty used across studies. One study^20^ reported that interspinous distance, pelvic inlet and the anteroposterior diameter of the mid‐plane were significantly associated with CRM positivity only in female patients, whereas in another study^16^ tumour size was associated with the rate of conversion to open surgery, and the sacrum–pubic diameter was associated with duration of surgery in male patients. In addition, the angle between the pubic symphysis and the sacral promontory was correlated to the quality of TME in laparoscopic LAR/ISR^21^, whereas the promontorium–subsacrum angle was documented as an independent predictor of CRM positivity in laparoscopic LAR^22^. Other measures of clinical importance were the sacral length, sacral angle and intertuberous distance^23^.

In a recent study^15^, BMI over 30 kg/m^2^, coloanal anastomosis, small intertuberous distance and high mesorectal fat tissue area were associated with surgical difficulty in TME; these variables were used to estimate the degree of surgical difficulty and categorize patients into low‐ or high‐grade groups with regard to surgical difficulty in the present study. Notably, in the present study, the local recurrence rate was significantly higher in the high‐grade than in the low‐grade group, indicating that the proposed surgical difficulty score could reflect the long‐term prognosis as well as intraoperative and short‐term postoperative outcomes.

Multivariable logistic regression analysis revealed that high BMI (above 25 kg/m^2^), large tumour size, large anorectal angle and short pelvic outlet were significantly associated with a high degree of surgical difficulty. However, the range of BMI in this series was lower than that of populations in Western countries, so the cut‐off value of BMI used in the present study (although in accordance with other experiences^17^, ^25^, ^26^) may not be reliable in a Western population, and other measures, such as the visceral or mesorectal fat area, may be more sensitive in predicting surgical difficulty^15^, ^27^. Tumour size is a well known risk factor for prolonged operating time, as well as for anastomotic leakage after rectal surgery^13^, ^16^, ^23^. Some previous studies^18^, ^28^ have reported the importance of pelvic outlet in rectal surgery in relation to duration of surgery and anastomotic leakage. A few studies^29^, ^30^, ^31^ showed that the anorectal angle is associated with defaecation, and can be used as a predictor for incontinence. Although it is not clear why anorectal angle was greater in the high‐grade surgical difficulty group, this association may be explained partially by the tonic activity of the puborectalis muscle and/or external sphincter muscle. It was also found in the present study that preoperative treatment (nCRT and NAC) resulted in a reduction in the anorectal angle as well as tumour size, which suggests that preoperative treatments may be useful in lessening the surgical difficulty by decreasing both tumour size and the anorectal angle.

Limitations of this study include its retrospective nature as well as its limited sample size (of patients undergoing laparoscopic and robotic procedures) from a single centre. Further investigation with larger cohorts in a relative short prospective period would help to validate these findings.

This case series included exclusively patients who had laparoscopic restorative procedures (LAR and ISR), because APR and other extended operations should be investigated separately due to differences in intraoperative and postoperative outcomes.

The present study found that surgical difficulty in minimally invasive rectal surgery was significantly associated with four variables (BMI, tumour size, anorectal angle and pelvic outlet). These factors should be taken into consideration when planning laparoscopic or robotic TME. If patients have a high score (3 or more), they should be regarded as having higher risk of a technically difficult operation.

## Supporting information


**Fig. S1** Effects of preoperative treatment on MRI‐related parameters

**Table S1** Surgical difficulty score
**Table S2** Association between clinical data and surgical difficulty criteria
**Table S3** Association between surgical difficulty and four risk factors
