# Supplementary material for: Prediction of surgical difficulty in minimally invasive surgery for rectal cancer by use of MRI pelvimetry
Source: BJS Open. 2020 Apr 28;4(4):666–77. doi: 10.1002/bjs5.50292 (PMC7397373; doi:10.1002/bjs5.50292)

**BJS5_50292**

**Prediction of surgical difficulty in minimally invasive surgery for rectal cancer by use of MRI pelvimetry**

**T. Yamamoto, K. Kawada, Y. Kiyasu, Y. Itatani, R. Mizuno, K. Hida and Y. Sakai**

**Table S1** Surgical difficulty score

**Table S2** Association between clinical data and surgical difficulty criteria

**Table S3** Association between surgical difficulty and four risk factors

**Fig. S1** Effects of preoperative treatment on MRI-related parameters

**A.** tumour size, **B.** tumour height from anal verge, **C.** anorectal angle, **D.** pelvic inlet, **E.** pubococcygeal distance, **F.** sacral depth, **G.** pelvic length, **H.** pelvic outlet, **I.** intertuberous distance, and **J.** interspinous distance

Medians; bars. (*, *P* < 0.05, Mann-Whitney U test).


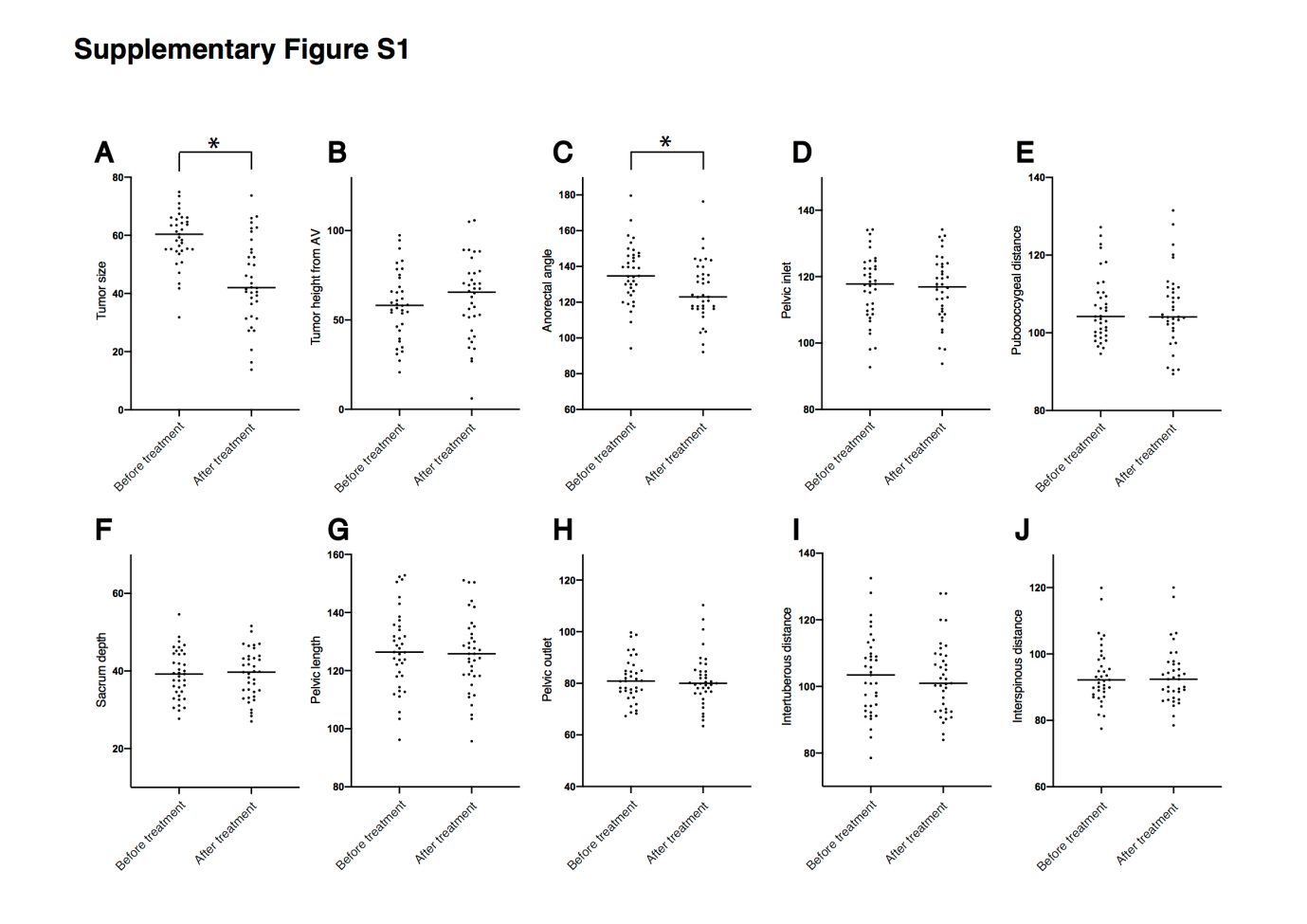

Supplement: Supplementary file 1 — Fig. S1 Effects of preoperative treatment on MRI‐related parameters [file BJS5-4-666-s001.docx]
