# Supplementary material for: Prediction of surgical difficulty in minimally invasive surgery for rectal cancer by use of MRI pelvimetry
Source: BJS Open. 2020 Apr 28;4(4):666–77. doi: 10.1002/bjs5.50292 (PMC7397373; doi:10.1002/bjs5.50292)
Supplement: Supplementary file 2 — Table S1 Surgical difficulty score Table S2 Association between clinical data and surgical difficulty criteria Table S3 Association between surgical difficulty and four risk factors [file BJS5-4-666-s002.pdf]

Supplementary Table S1. Surgical difficulty score

Surgical Difficulty Score	Points
Operation time >350 min	3
Conversion to open surgery	3
Transanal approach	2
Postoperative hospital stay >18 days	2
Blood loss >100 ml	1
Morbidity ≥ Grade II	1

Supplementary Table S2. Association between clinical data and surgical difficulty criteria

	**Sex**		
	Male	Female	*P*
Operation time (min)			
>350; ≤350	34;48	6;33	**0.004***
Conversion to open surgery			
Yes; No	2;80	1;38	1.000
Postoperative hospital stay (day)			
>18; ≤18	43;39	12;27	**0.025***
Blood loss (ml)			
>100; ≤100	21;61	2;37	**0.006***
Morbidity			
Grade 0–1; 2–3	60;22	35;4	**0.038***

	**BMI**		
	≤25	>25	*P*
Operation time (min)			
>350; ≤350	26;72	14;9	**0.002***
Conversion to open surgery			
Yes; No	2;96	1;22	0.472
Postoperative hospital stay (day)			
>18; ≤18	40;58	15;8	**0.034***
Blood loss (ml)			
>100; ≤100	14;84	9;14	**0.006***
Morbidity			
Grade 0–1; 2–3	80;18	15;8	0.085

	**Age**		
	<65	≥65	*P*
Operation time (min)			
>350; ≤350	22;44	18;37	0.944
Conversion to open surgery			
Yes; No	2;64	1;54	1.000
Postoperative hospital stay (day)			
>18; ≤18	30;36	25;30	1.000
Blood loss (ml)			
>100; ≤100	13;53	10;45	0.833
Morbidity			
Grade 0–1; 2–3	52;14	43;12	0.936

	**Tumor location**		
	Low	High	*P*
Operation time (min)			
>350; ≤350	22;28	18;53	**0.032***
Conversion to open surgery			
Yes; No	2;48	1;70	0.569
Postoperative hospital stay (day)			
>18; ≤18	27;23	28;43	0.113
Blood loss (ml)			
>100; ≤100	12;38	11;60	0.240
Morbidity			
Grade 0–1; 2–3	40;10	55;16	0.738

	**Surgery type**		
	LAR	ISR	*P*
Operation time (min)			
>350; ≤350	33;74	7;7	0.152
Conversion to open surgery			
Yes; No	3;104	0;14	1.000
Postoperative hospital stay (day)			
>18; ≤18	47;60	8;6	0.350
Blood loss (ml)			
>100; ≤100	20;87	3;11	0.729
Morbidity			
Grade 0–1; 2–3	81;26	14;0	**0.037***

	**Surgical technique**		
	Lap	Robot	*P*
Operation time (min)			
>350; ≤350	31;73	9;8	0.060
Conversion to open surgery			
Yes; No	3;101	0;17	1.000
Postoperative hospital stay (day)			
>18; ≤18	49;55	6;11	0.364
Blood loss (ml)			
>100; ≤100	18;86	5;12	0.314
Morbidity			
Grade 0–1; 2–3	82;22	13;4	0.760

Supplementary Table S3. Association between surgical difficulty and 4 risk factors

Combinations of 4 risk factors	Low-grade, %(n)	High-grade, %(n)
None	100 (15/15)	0 (0/15)
BMI > 25	65 (15/23)	35 (8/23)
Tumor size > 45	80 (47/60)	20 (13/60)
Anorectal angle > 123	77 (47/61)	23 (14/61)
Pelvic outlet < 82.7	83 (50/60)	17 (10/60)
BMI > 25 + Tumor size > 45	50 (6/12)	50 (6/12)
BMI > 25 + Anorectal angle > 123	50 (7/14)	50 (7/14)
BMI > 25 + Pelvic outlet < 82.7	33 (3/9)	67 (6/9)
Tumor size > 45 + Anorectal angle > 123	70 (23/33)	30 (10/33)
Tumor size > 45 + Pelvic outlet < 82.7	76 (22/29)	24 (7/29)
Anorectal angle > 123 + Pelvic outlet < 82.7	72 (21/29)	28 (8/29)
BMI > 25 + Tumor size > 45 + Anorectal angle > 123	29 (2/7)	71 (5/7)
BMI > 25 + Tumor size > 45 + Pelvic outlet < 82.7	0 (0/4)	100 (4/4)
BMI > 25 + Anorectal angle > 123 + Pelvic outlet < 82.7	17 (1/6)	83 (5/6)
Tumor size > 45 + Anorectal angle > 123 + Pelvic outlet < 82.7	67 (10/15)	33 (5/15)
All	0 (0/3)	100 (3/3)
